# Innominate Vein Thrombosis: A Case Report and Literature Review

**DOI:** 10.7759/cureus.64145

**Published:** 2024-07-09

**Authors:** Nikola Stojanovic, Emmanuel Ukenenye, Asma Syed

**Affiliations:** 1 Medicine, Brookdale University Hospital and Medical Center, Brooklyn, USA; 2 Internal Medicine, Brookdale University Hospital and Medical Center, Brooklyn, USA; 3 Electrophysiology and Cardiology, Brookdale University Hospital and Medical Center, Brooklyn, USA

**Keywords:** thrombosis, brachiocephalic vein, innominate vein, breast cancer, implantable cardioverter-defibrillator (icd)

## Abstract

The brachiocephalic vein (BCV), also known as the innominate vein, is a central vein in the upper chest formed by merging the internal jugular and subclavian veins. It plays a crucial role in venous return from the head, neck, and upper extremities and is significant in procedures such as pacemaker and implantable cardioverter-defibrillator (ICD) placement, chemotherapy ports, and central venous catheter insertions. The presence of foreign bodies and local malignancy are major risk factors for thrombosis in the BCV. As part of the deep venous system, BCV thrombosis (BCVT) is a rare condition but can lead to serious complications like superior vena cava syndrome and, rarely, pulmonary embolism. This case report presents an 82-year-old woman with a history of heart failure with reduced ejection fraction, coronary artery disease, atrial fibrillation, HIV, pulmonary embolism, systemic lupus erythematosus, and breast cancer who required an ICD placement due to persistent systolic dysfunction. During the procedure, chronic BCVT leading to the stenosis was incidentally discovered, necessitating urgent vascular intervention to establish venous patency. The patient’s complex medical history, including previous chemotherapy through a central venous catheter, contributed to the risk factors for BCVT. The multidisciplinary approach led to successful ICD placement and the reinstatement of anticoagulation therapy. This case underscores the rarity and severity of BCVT and highlights the importance of pre-procedural imaging, such as CT venography, in patients with multiple risk factors. Additionally, the report suggests considering leadless ICD technology for patients with limited venous access to avoid complications. The findings emphasize the critical need for thorough evaluation and planning in complex cases to ensure successful outcomes.

## Introduction

The brachiocephalic vein (BCV), also known as the innominate vein, is a central vein in the upper chest that rises by the merging of internal jugular and subclavian veins on each side of the neck, and it plays a crucial role in venous return from the head, neck, and upper extremities. The right and left BCVs converge to form the superior vena cava (SVC). Their clinical significance also lies in the accessibility of BCV in multiple procedures, such as a pacemaker, implantable cardioverter-defibrillator (ICD), chemotherapy port, and central venous catheter placement [1]. The presence of foreign bodies in the BCV and local malignancy are significant risk factors for thrombosis formation. As a part of the deep venous system, BCV thrombosis (BCVT) is a rare condition associated with a risk of SVC syndrome and pulmonary embolism [2].

This case report presents an 82-year-old woman with a previous medical history of heart failure with reduced ejection fraction, single-vessel coronary artery disease treated with a drug-eluting stent in the left descending artery (16 months ago), atrial fibrillation, HIV, history of pulmonary embolism, systemic lupus erythematosus, and positive oncologic history (breast cancer) who came for a planned ICD due to a lack of improvement of systolic heart function despite quadruple guideline-directed medical therapy for more than six months, resulting in an incidental finding of BCVT and its treatment through the multidisciplinary approach.

## Case presentation

The patient, an 82-year-old woman with a history of right breast invasive ductal carcinoma in situ (estrogen receptor positive and Her2 negative), underwent successful treatment including lumpectomy, radiation therapy, and adjuvant chemotherapy (four cycles of doxorubicin-cyclophosphamide and 12 weeks of paclitaxel via a right-sided internal jugular vein tunneled central venous catheter). She regularly followed up with her oncologist and received anti-estrogen therapy with 1 mg of anastrozole daily for recurrence prevention.

**Figure 1:**
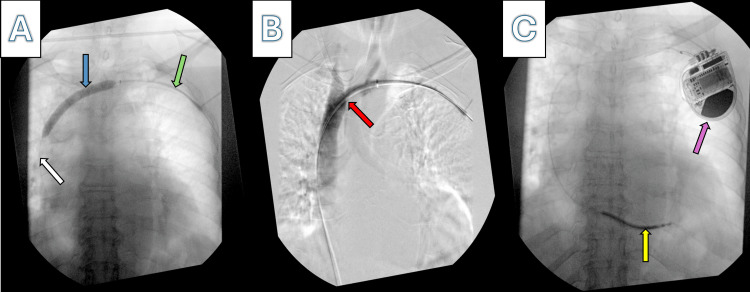
Innominate/BCVT on venography (A) The guidewire in the left subclavian vein passed through the stenotic left BCV up to the SVC, which enabled the successful balloon dilatation of the stenotic region (white arrow: the distal part of the guidewire in the SVC; blue arrow: balloon dilatation of the left BCV; green arrow: guidewire in the left subclavian vein). (B) Venography shows that the contrast passes through the left subclavian and left BCVs and down to the SVC, thus confirming the patency of the central veins (red arrow: contrast passes through the BCV after patency is reestablished). (C) The successful placement of the ICD (yellow arrow: lead of the successfully placed ICD; purple arrow: the generator of the ICD). BCV, brachiocephalic vein; BCVT, brachiocephalic vein thrombosis; ICD, implantable cardioverter-defibrillator; SVC, superior vena cava

Before surgery, she received 2 g of intravenous cefazolin for preoperative prophylaxis. General anesthesia induction with intravenous propofol was conducted under continuous cardiovascular monitoring and pulse oximetry. Intraoperatively, the electrophysiology team encountered difficulty advancing the wire in the left axillary vein due to high-grade stenosis of the left innominate vein, as confirmed by venography shown in Figure 1.

The vascular surgery team was summoned for assistance due to difficulties placing the leads caused by vein stenosis from thrombosis, necessitating invasive procedures to restore vein patency. Initially, a 6F sheath was followed by a 7F sheath to enable the guidewire to navigate the high-grade stenosis of the left innominate vein. The stenosis was due to an old, organized thrombus that could not be mechanically removed. Subsequently, balloon dilatation of the stenotic left innominate vein was performed. Post-vascular intervention venography confirmed patency, allowing the electrophysiology team to proceed with further ICD placement.

The ICD was successfully implanted without complications. The patient resumed therapeutic direct oral anticoagulation therapy (apixaban 5 mg twice daily) considering her history of atrial fibrillation, a prior episode of pulmonary embolism, and BCVT. The following day, she was discharged home for outpatient follow-up with cardiology, oncology, and primary care physicians.

## Discussion

In the 19th century, the German physician Rudolf Virchow postulated three critical components of thrombosis, also known as Virchow’s triad, which are endothelial injury, stasis or abnormal blood flow, and hypercoagulability [3]. In the literature, innominate/BCVT documentation is limited. A study of hospitalized patients over the age of 20 noted thromboembolic pathology of the BCVs, or SVC, in <1% of the study population [1,2].

Innominate vein thrombosis, or BCVT, is rare but severe. BCVT is categorized as a form of deep vein thrombosis (DVT), which has a higher prevalence in the lower limbs but, in some instances, affects the upper limbs. BCVT is classified under upper extremity DVT, which comprises thrombosis in the arms, shoulders, and upper chest veins. The predisposing risk factors for BCVT are similar to those for other forms of DVT [4]. Several factors can contribute to its development, such as active malignancy in its proximity, foreign objects (central venous catheters, pacemaker wires, and ICD), hypercoagulable state, local trauma or surgery, local compression (thoracic outlet syndrome), intravenous drug use, and rare medical conditions such as Behçet’s disease, thoracic aortic aneurysms, or mediastinal fibrosis.

For instance, a tumor thrombus in the BCV extending into the SVC has been observed in a patient with papillary thyroid cancer [5]. In a retrospective study of patients hospitalized with thrombosis of the BCV and SVC, almost 75% had cancer, and 65% had central venous access lines. Most of the patients were symptomatic and experienced nuchal pain or discomfort and facial, nuchal, and arm edema [1].

Clinical manifestations

BCVT can vary from asymptomatic, like in our case report and the majority of the patients, to symptomatic. The frequency with which it is symptomatic depends on several factors, including the thrombus’s size, its development’s rapidity, and individual patient characteristics. If symptoms occur, the most common are swelling, pain, and discoloration in the neck, face, and upper extremities. The severity of symptoms correlates with the extent of thrombosis and whether it causes significant obstruction to venous blood flow [6].

The proportion of symptomatic versus asymptomatic cases in innominate vein thrombosis is not precisely defined in the medical literature, mainly due to the rarity of this condition. In cases where the thrombosis develops slowly, the body might have time to create collateral circulation, which can reduce the severity of symptoms.

Diagnosis

Diagnosing innominate vein thrombosis, like other types of venous thrombosis, typically involves clinical assessment and imaging studies. Suggestive clinical presentation and predisposing history should prompt imaging studies to prove the BCVT. The initial imaging is usually Doppler ultrasound due to its portability and accessibility. Computed tomography, or magnetic resonance venography, is usually used if the ultrasound imaging is inconclusive or if more detailed imaging is necessary. Sometimes, the diagnosis is made incidentally during the invasive procedure, like in our case report, using venography and failing to advance the wire [7].

Complications

The complications primarily arise from the obstruction of blood flow and the potential for clot migration. The incidence and prevalence of these complications need to be better established in the literature due to the rarity of this thrombotic disease. BCVT can cause several complications, some of which can be life-threatening, as outlined in Table 1.

**Table 1:** Complications of BCVT BCVT, brachiocephalic vein thrombosis; DVT, deep vein thrombosis; PE, pulmonary embolism; PTS, post-thrombotic syndrome; SVC, superior vena cava; SVCS, superior vena cava syndrome

Complication	Description
SVCS	An oncologic emergency that results in swelling and cyanosis of the face, neck, and upper chest associated with shortness of breath, dizziness, and headache. The BCVT can extend into the SVC, resulting in more extensive venous congestion [8].
PE	Despite its proximity to the pulmonary circulation, it has a lower chance of causing PE (<10%) than DVT of the lower extremities. The reasons why PE is less common in BCVT are likely differences in gravitational factors between both the upper and lower extremities, venous flow hemodynamics such as more sluggish blood flow in the lower extremities, clot size that tends to be more prominent in the lower extremities, and a more robust collateral venous system enabling blood flow around the clot, minimizing the probability of thrombus breakage [9].
Local infections, recurrence of thrombosis, and phlebitis	Due to venous stasis, blood accumulates and irritates the vascular lining, leading to inflammation, clot formation, extravasation, and infection [10].
PTS	A long-term complication that occurs after DVT and encompasses chronic pain, swelling, and sometimes skin integrity breakage of the affected area. However, documentation of PTS’s association with BCVT is scarce [11].

Treatment options

As shown in Table 2, several treatment options exist for BCVT, such as thrombolysis, interventional measures, anticoagulant therapy, and treating the background etiology or risk factor [12]. To stop BCVT from recurring, finding and addressing its underlying cause is essential. Thoracic outlet syndrome, cancer, central venous catheterization, and hypercoagulable diseases are common causes or risk factors. Treatments that may be specific include surgical decompression, long-term anticoagulation in situations of hypercoagulable illnesses, the removal of the catheter, and optimal management of malignancies [12].

**Table 2:** Treatment of BCVT BCVT, brachiocephalic vein thrombosis; DOAC, direct-acting oral anticoagulant; LMWH, low molecular weight heparin

Treatment	Description
Anticoagulant	The primary treatment for BCVT is to prevent new clot formation and facilitate spontaneous clot breakdown. Warfarin is less frequently used than LMWH. Recurrence risk and the underlying risk factor will determine the duration of anticoagulants, usually for three to six months. Some studies have reported acceptable safety and efficacy with DOACs. Specific DOACs were not evaluated separately [13,14].
Thrombolysis and interventional procedures	In cases of BCVT with a high clot burden, significant symptoms, or imminent limb ischemia, it is typically taken into consideration. Catheter-directed thrombolysis is preferred. Because there is a chance of bleeding issues, this procedure must be closely monitored [15].

Our case

In our case, the patient developed vein stenosis due to chronic venous thrombosis, influenced by risk factors including a history of a right-sided tunneled catheter for chemotherapy, a history of breast cancer, and systemic lupus erythematosus. Given the need for ICD placement, it was necessary to establish the patency of the BCV by sequentially dilating the venous stenosis to allow the ICD leads to pass through to the right side of the heart. This procedure was successful on the first attempt.

For patients with limited central venous access who require an ICD, the subcutaneous ICD would represent a cutting-edge technology that offers a viable alternative [16].

Our patient was taking a direct oral anticoagulant, apixaban 5 mg twice daily, due to a previous history of atrial fibrillation and pulmonary embolism, and was notably compliant with the medication. We hypothesize that in our case, the presence of the foreign body in the central veins was a significant pro-thrombotic risk factor leading to chronic venous thrombosis, which could have been mitigated but not fully prevented by anticoagulant therapy. Alternatively, the BCVT may have developed slowly before the initiation of anticoagulant therapy for atrial fibrillation and pulmonary embolism, with the nearby tumor acting as a pro-thrombotic nidus. Unfortunately, our hypotheses cannot be proven and represent the only reasonable explanations the medical team could think of.

The significance of this case report lies in the rarity of thrombosis in this location, the complexity of our case due to multiple risk factors for BCVT, the absence of clinical symptoms, and the review of current management options. In cases where patients have multiple risk factors, it is rational to consider CT venography before ICD placement to avoid situations like the one in our report. If venography reveals stenosis of the central veins, the newer option of a leadless ICD could be considered to avoid lead-associated complications [16].

## Conclusions

This case report underscores the significance of the rarity of BCVT, the lack of clinical signs or symptoms, and the presence of multiple risk factors, such as a tunneled central venous catheter for chemotherapy, a history of cancer, and systemic lupus erythematosus. The incidental finding of BCV stenosis due to chronic thrombosis in our patient necessitated urgent vascular surgery to establish vein patency, leading to the successful placement of an ICD. This report highlights the importance of addressing this rare presentation and suggests that CT venography should be considered to assess central venous anatomy before ICD or pacemaker placement in patients with multiple risk factors for central venous thrombosis. Additionally, the use of leadless ICD technology should be considered in cases with limited venous anatomy to avoid lead-associated complications. Our findings emphasize the need for a thorough pre-procedural evaluation in complex cases to ensure optimal patient outcomes.

## References

[1] Otten TR, Stein PD, Patel KC, Mustafa S, Silbergleit A (2003). Thromboembolic disease involving the superior vena cava and brachiocephalic veins. Chest.

[2] Oymak FS, Buyukoglan H, Tokgoz B (2005). Prevalence of thromboembolic disease including superior vena cava and brachiocephalic veins. Clin Appl Thromb Hemost.

[3] Lowe GD (2003). Virchow's triad revisited: abnormal flow. Pathophysiol Haemost Thromb.

[4] Kucher N (2011). Deep-vein thrombosis of the upper extremities. N Engl J Med.

[5] Koike E, Yamashita H, Watanabe S, Yamashita H, Noguchi S (2002). Brachiocephalic vein thrombus of papillary thyroid cancer: report of a case. Surg Today.

[6] Grigorian A, Nahmias JT (2024). Upper extremity deep venous thrombosis. StatPearls [Internet].

[7] Heil J, Miesbach W, Vogl T, Bechstein WO, Reinisch A (2017). Deep vein thrombosis of the upper extremity: a systematic review. Dtsch Arztebl Int.

[8] Rice TW, Rodriguez RM, Light RW (2006). The superior vena cava syndrome: clinical characteristics and evolving etiology. Medicine (Baltimore).

[9] Tapson VF (2008). Acute pulmonary embolism. N Engl J Med.

[10] Joffe HV, Goldhaber SZ (2002). Upper-extremity deep vein thrombosis. Circulation.

[11] Ginsberg JS (1996). Management of venous thromboembolism. N Engl J Med.

[12] Kearon C, Akl EA, Ornelas J (2016). Antithrombotic therapy for VTE disease: CHEST guideline and expert panel report. Chest.

[13] Montiel FS, Ghazvinian R, Gottsäter A, Elf J (2017). Treatment with direct oral anticoagulants in patients with upper extremity deep vein thrombosis. Thromb J.

[14] Porfidia A, Agostini F, Giarretta I (2020). Upper extremity deep vein thrombosis treated with direct oral anticoagulants: a multi-center real world experience. J Thromb Thrombolysis.

[15] Vik A, Holme PA, Singh K, Dorenberg E, Nordhus KC, Kumar S, Hansen JB (2009). Catheter-directed thrombolysis for treatment of deep venous thrombosis in the upper extremities. Cardiovasc Intervent Radiol.

[16] Kaya E, Rassaf T, Wakili R (2019). Subcutaneous ICD: current standards and future perspective. Int J Cardiol Heart Vasc.

